# Effectiveness of the Offer of the Smoke Free Smartphone App Compared With No Intervention for Smoking Cessation: Pragmatic Randomized Controlled Trial

**DOI:** 10.2196/50963

**Published:** 2024-11-15

**Authors:** Sarah Jackson, Dimitra Kale, Emma Beard, Olga Perski, Robert West, Jamie Brown

**Affiliations:** 1 Department of Behavioural Science and Health University College London London United Kingdom

**Keywords:** randomized controlled trial, smartphone app, smoking cessation, digital intervention, tobacco, mobile phone

## Abstract

**Background:**

Digital technologies offer the potential for low-cost, scalable delivery of interventions to promote smoking cessation.

**Objective:**

We aimed to evaluate the effectiveness of the offer of Smoke Free—an evidence-informed, widely used app—for smoking cessation versus no support.

**Methods:**

In this 2-arm randomized controlled trial, 3143 motivated adult smokers were recruited online between August 2020 and April 2021 and randomized to receive an offer of the Smoke Free app plus follow-up (intervention arm) versus follow-up only (comparator arm). Both groups were shown a brief message at the end of the baseline questionnaire encouraging them to make a quit attempt. The primary outcome was self-reported 6-month continuous abstinence assessed 7 months after randomization. Secondary outcomes included quit attempts in the first month post randomization, 3-month continuous abstinence assessed at 4 months, and 6-month continuous abstinence at 7 months among those who made a quit attempt. The primary analysis was performed on an intention-to-treat (ITT) analysis basis. Sensitivity analyses included (1) restricting the intervention group to those who took up the offer of the app, (2) using complete cases, and (3) using multiple imputation.

**Results:**

The effective follow-up rate for 7 months was 41.9%. The primary analysis showed no evidence of a benefit of the intervention on rates of 6-month continuous abstinence (intervention 6.8% vs comparator 7.0%; relative risk 0.97, 95% CI 0.75-1.26). Analyses of all secondary outcomes also showed no evidence of a benefit. Similar results were observed on complete cases and using multiple imputation. When the intervention group was restricted to those who took up the offer of the app (n=395, 25.3%), participants in the intervention group were 80% more likely to report 6-month continuous abstinence (12.7% vs 7.0%; relative risk 1.80, 95% CI 1.30-2.45). Equivalent subgroup analyses produced similar results on the secondary outcomes. These differences persisted after adjustment for key baseline characteristics.

**Conclusions:**

Among motivated smokers provided with very brief advice to quit, the offer of the Smoke Free app did not have a detectable benefit for cessation compared with follow-up only. However, the app increased quit rates when smokers randomized to receive the app downloaded it.

**Trial Registration:**

ISRCTN ISRCTN85785540; https://www.isrctn.com/ISRCTN85785540

**International Registered Report Identifier (IRRID):**

RR2-https://onlinelibrary.wiley.com/doi/full/10.1111/add.14652

## Introduction

Tobacco remains one of the leading causes of disease and preventable death worldwide, killing more than 8 million people each year [1]. A range of evidence-based support options are available to help smokers quit [2-4], but barriers including a lack of time and (in countries where free support is unavailable) inability to pay mean that many smokers do not access this support [5-7]. Digital technologies offer the potential for low-cost, scalable delivery of interventions to promote smoking cessation. More smokers have turned to remote options for cessation support since the COVID-19 pandemic began, including smartphone apps [8], but evidence of the effectiveness of existing apps is lacking. This study aimed to assess the effectiveness of the offer of a widely used app for smoking cessation compared with no support.

Digital support for smoking cessation has the potential to contribute to meaningful reductions in smoking prevalence in countries around the world. In particular, the past decade has seen a surge in smartphone apps offering support for smokers who want to quit [9]. Combined with increasing levels of smartphone ownership (currently estimated at 3.8 billion worldwide [10]; 74% of the adult population [11]), these apps can reach large numbers of smokers. However, the potential of apps for promoting cessation is not yet being realized. A 2019 Cochrane review of mobile phone interventions for smoking cessation [12] identified just 5 studies (total N=3079) that compared a smoking cessation smartphone app with lower-intensity smoking cessation support. The pooled data provided no evidence that apps increased the likelihood of cessation (relative risk [RR] 1.00, 95% CI 0.66-1.52), but the evidence was judged to be of very low certainty limiting confidence in the effect estimate. The authors called for more large-scale randomized controlled trials (RCTs) to establish whether smartphone app interventions are effective for smoking cessation. In 2020, a large RCT [9] (n=2415) tested the efficacy of a smoking cessation app based on acceptance and commitment therapy compared with a simpler app informed by US clinical practice guidelines. Self-reported quit rates at 12 months were higher among participants randomized to use the acceptance and commitment therapy–based app (28.2% vs 21.1%, odds ratio 1.49, 95% CI 1.22-1.83). To our knowledge, no published RCTs have compared apps designed to provide ongoing support with unaided quitting.

With over 6 million downloads to date and 70,000 new users each month, Smoke Free is one of the world’s most widely used smoking cessation apps. The app is evidence-informed and available for iOS and Android OS. In a large exploratory RCT (n=28,112) conducted between 2013 and 2015, the full version of the app increased 3-month self-reported continuous abstinence rates compared with a reduced version (odds ratio 1.90, 95% CI 1.53-2.37) [13]. However, this result was limited by relatively short-term outcomes and very low follow-up rates (8.5% and 6.5% in the intervention and control conditions, respectively) with no active attempts to recontact participants. Smoke Free therefore constitutes a useful test bed for assessing the effectiveness of a smartphone app for smoking cessation versus unaided quitting.

This paper describes the results of the App for Smoking Cessation Evaluation Trial [14], a 2-arm RCT designed to evaluate the effectiveness of the offer of the Smoke Free app in increasing rates of tobacco smoking cessation compared with follow-up only. We adopted a pragmatic design to provide information on the usefulness of this app in real-world settings. Given that, in an effectiveness trial of this nature, not all participants who are offered the intervention will take it up, we were therefore testing the offer rather than the actual use of the app. The primary research question was:

1. How effective is an offer to use the app plus follow-up (intervention) compared with no offer of the app and follow-up only (comparator) in promoting self-reported smoking cessation for at least 6 months, assessed 7 months after enrollment?

We also addressed several secondary research questions:

1. How effective is the intervention in promoting:
  At least one quit attempt in the 1 month following enrollment in this study?
  Smoking cessation for at least 3 months, assessed 4 months after enrollment?
  Smoking cessation for at least 6 months among those who make at least one quit attempt in the 1 month following enrollment in this study, assessed 7 months after enrollment?
  Downloading or using the Smoke Free app at least once, assessed 7 months after enrollment?
2. Do the answers to the primary research question and secondary research questions differ according to smokers’ gender, level of cigarette addition, age, education, financial situation, or prior experience with a smoking cessation smartphone app?

## Methods

### Overview

A summary timeline of trial procedures is shown in Multimedia Appendix 1.

### Design

The app for the smoking cessation evaluation trial was a 2-arm individual RCT. This study’s protocol [14] and analysis plan [15] were preregistered (ISRCTN85785540). An independent Trial Steering Committee provided overall supervision of the trial.

### Setting

This study was conducted online using the Qualtrics survey platform, with no restriction on location.

### Participants

Inclusion criteria were current cigarette smoker, aged ≥18 years, English speaker, owns a smartphone, provided a valid email address not previously used by another participant, interested in making a quit attempt within the next month, and willing to be followed up by email and complete online questionnaires.

Eligibility was assessed via screening questions embedded at the start of the baseline study questionnaire on Qualtrics. Those who did not meet the inclusion criteria were directed to the NHS Smokefree website [16] for resources to help with quitting smoking.

Participants were not provided with any financial compensation, but we offered to donate £10 (US $12) to a cancer charity on behalf of each participant who responded to the final follow-up.

### Sample Size

The intended sample size was decided a priori based on achieving 90% power to detect a RR ≥1.5 with an α of *P*<.05, 1‐tailed, and a quit rate of 6.0% in the comparator group. This led to a target sample size of 3116; 1558 per group.

We note that we amended our original power calculation as reported in the published protocol [14]. Details of these amendments and the rationale underlying them are available on the Open Science Framework [15]. The final sample size target was approved by the Trial Steering Committee on January 6, 2021, and registered on ISRCTN (International Standard Randomised Controlled Trial Number) in January 2021 after we had randomized 2798 participants and before any of the primary outcome data (6-month continuous abstinence assessed at 7 months) were collected.

### Ethical Considerations

Ethical approval for the trial was obtained from the University College London Research Ethics Committee (CEHP/2020/579). All participants were provided with a summary of this study and their right to withdraw on the landing page of the baseline survey on Qualtrics. They provided informed consent by selecting “Yes: I confirm I have read the information about the study and wish to participate.”

### Recruitment

Recruitment took place between August 2020 and April 2021. Participants were recruited via advertisements on social media (Facebook and Twitter; Multimedia Appendix 1) and a mailing list of smokers who had previously signed up for the Smoke Free app and had agreed to be contacted. We emailed people on the mailing list who signed up to the app >6 months previously with an invitation to participate in this study. Response to these emails was low (~5/1000). Most of our participants were recruited via Facebook adverts (at an average cost of £3 [US $3.92]/participant), which linked to the Qualtrics baseline questionnaire (with embedded consenting procedure and screening questions).

### Randomization

Consenting participants who met the eligibility criteria were randomized after completing the baseline questionnaire. Randomization was 1:1 at the individual level with no restriction (ie, no blocking) and was automated within Qualtrics, such that each participant was shown at random either the intervention message including the offer of the Smoke Free app or the comparator message after the final questionnaire question.

All investigators were blinded to participants’ treatment allocation until all data had been collected. The data were analyzed blindly by the trial statistician (EB).

### Interventions

#### Comparator

After consenting and completing the baseline questionnaire, participants in the comparator condition were shown a final screen with a brief message encouraging them to make a quit attempt within the next 4 weeks and reminding them of the importance of responding to follow-up requests designed to track their progress (Multimedia Appendix 1). This same message was also emailed to them immediately afterward.

#### Intervention: Smoke Free App

Participants in the intervention condition received the same advice as those in the comparator condition plus the offer of the full version of the Smoke Free app free of charge, encouragement to use the app, and a link to download it. This same message and information on how to access the app were also emailed to them immediately afterward.

The Smoke Free app is based on behavior change techniques that would be expected from theory [13] and evidence with face-to-face support [17,18] to aid smoking cessation. It guides smokers through the first 3 months of their quit attempt by helping them maintain their resolve by setting a clear goal, monitoring their progress toward that goal, and becoming aware of the benefits of being smoke-free achieved to date. More detail is provided in Multimedia Appendix 1.

### Follow-Up Data Collection

Follow-up data was collected between September 2020 and December 2021, via online questionnaires 1, 4, and 7 months after study enrollment. Invitations to complete the 1- and 4-month surveys were sent via email (automated within Qualtrics). For the final (7-month) follow-up, to boost response rates for our primary outcome, we contacted participants up to 6 times over 2 weeks. First, they were invited via email within Qualtrics. Next, a further email invitation was sent from one of the research team’s personal email addresses (to reduce the “spam” rating of the email). Then participants who provided their phone numbers were contacted via SMS text messaging (between March and August 2021) or telephone call (between August and December 2021; a change implemented to boost the response rate) and asked to respond “yes” or “no” to the key outcome assessment. Finally, up to 3 further emails were sent asking the same question as the SMS text messaging, prompting participants to a direct “yes” or “no” response via email. Participants who responded to any of the invitations or reminders were not contacted further.

### Measures

#### Participant Baseline Characteristics

The baseline questionnaire assessed the following: email address, mobile phone number (optional), smartphone ownership, motivation to quit in the next month (Motivation To Stop Scale [19]), willingness to complete online questionnaires after 1, 4 and 7 months, age (18-34, 35-64, or ≥65 years), gender (male or female), education (any or no educational qualifications gained aged ≥16 years), financial status (live comfortably, meet needs with a little left, just meet basic expenses, or do not meet basic expenses) [20], country of residence, first language (English or other), number of cigarettes smoked per day, level of cigarette addiction (first cigarette after waking within 5 minutes, 6-30 minutes, 31-60 minutes, or >60 minutes), history of serious quit attempts (never or yes, not in the past year, or yes in the past year), and past and current use of support for smoking cessation (prescription nicotine replacement therapy or nicotine replacement therapy bought over-the-counter, varenicline, bupropion, face-to-face behavioral support, telephone support, written self-help materials, websites, or apps). In an exploratory addition to outcome assessment, given evidence that heart rate declines substantially when smokers stop [21], participants who had a heart rate monitoring device (eg, FitBit or Apple watch) were asked to report their average resting heart rate as measured by this device.

#### Outcomes

The primary outcome was the percentage of participants reporting not having smoked for 6 months at the 7-month follow-up. This was assessed in the online questionnaire with the question: “Have you smoked any cigarettes in the past 6 months?” with response options “none at all,” “between 1 and 5,” and “more than 5.” In line with the Russell Standard for self-report of smoking abstinence [22], the former 2 responses were collapsed for analysis, with data coded 1 for respondents reporting smoking no more than 5 cigarettes in the past 6 months and 0 for those reporting smoking more than 5 cigarettes. Where participants did not respond to the invitations to complete the questionnaire, the question was simplified to “Have you smoked more than 5 cigarettes in the past 6 months?” and participants were asked to reply “yes” or “no” via SMS text messaging, telephone, or email. Based on the intention-to-treat (ITT) analysis principle, those who did not respond to follow-up attempts were retained in the analyses and classified as continuing smokers [22].

Secondary outcomes were the percentage of participants reporting (1) quit attempts at 1-month follow-up, defined as having made a serious quit attempt in the last 4 weeks (assessed with the question: “Have you made a serious attempt to quit smoking in the last 4 weeks? Please include any attempt that you are currently making [yes=1/no=0],” (2) smoking cessation for at least 3 months at the 4-month follow-up (assessed with the question: “Have you smoked a single puff on a cigarette in the past 3 months? [yes=0/no=1]”), (3) smoking cessation for at least 6 months at the 7-month follow-up in those who made a quit attempt (assessed with the question: “Have you smoked any cigarettes in the past 6 months? [none at all=1/between 1 and 5=1/more than 5=0]”), and (4) app use, defined as downloading or using the Smoke Free app at least once at any point during this study’s period (assessed at the 7-month follow-up with the question: “In the last 7 months, have you downloaded or used the Smoke Free app (pictured) at least once? [yes=1/no=0]”).

### Statistical Analyses

#### Overview

We followed our preregistered analysis plan [14], with 2 amendments registered on the Open Science Framework before running the analysis [15] and 2 unplanned sensitivity analyses after running the analyses. Details of amendments to the analysis plan are summarized in Multimedia Appendix 2.

All variables were collected primarily online and entered automatically into a Qualtrics database. From this database, a user-specified Excel (Microsoft Corp) file was downloaded, subjected to basic processing and recoding, and integrated with responses provided by text messages. On completion, data were analyzed blind to intervention allocation using R Studio (version 4.2.1; R Foundation).

#### Primary Analyses

Our primary analyses used an ITT analysis approach, which treated those with missing data as smoking [22]. We used log-binomial regression to calculate the RR and 95% CI of each primary and secondary outcome in the intervention group versus the comparator group.

#### Moderation Analyses

For each primary and secondary end point, we ran a series of log-binomial regression models in which we added 2-way interactions between group and gender, cigarette addiction, age (<35/≥35 years to broadly distinguish between younger and older participants with the specific threshold corresponding to the age at which quitting avoids most of the excess mortality from smoking [23]), education, financial situation, and previous experience with using a smoking cessation app (based on self-reported use ever of cessation aids at baseline). The results are reported in Multimedia Appendix 3.

#### Sensitivity Analyses

We repeated our primary and secondary analyses (1) restricting the intervention group to participants who took up the offer of free full access to the Smoke Free app, which was self-reported or verified by matching the email address used to log in to the app to the one provided in the baseline questionnaire (with and without adjustment for key baseline characteristics); (2) restricting both groups to participants who were successfully followed up; and (3) using multiple imputation to impute missing outcomes data. In an exploratory analysis, we also repeated the analyses defining successful quits as self-reported abstinence plus a reduction in mean resting heart rate of ≥5 beats per minute, based on the lower 95% CI for the difference in resting heart rate between people smoking as usual and not smoking in a previous study [24]. Finally, we reanalyzed our primary outcome assuming different rates of abstinence in those not followed up on.

To aid interpretation of the strength of evidence for associations, we calculated Bayes factors [25] to differentiate between evidence for an effect, no effect, and data insensitivity. We used a half-normal distribution, the mode at 0 (no effect), and the SD equal to the expected effect size used in the sample size calculation (RR 1.5).

## Results

### Overview

**Figure 1:**
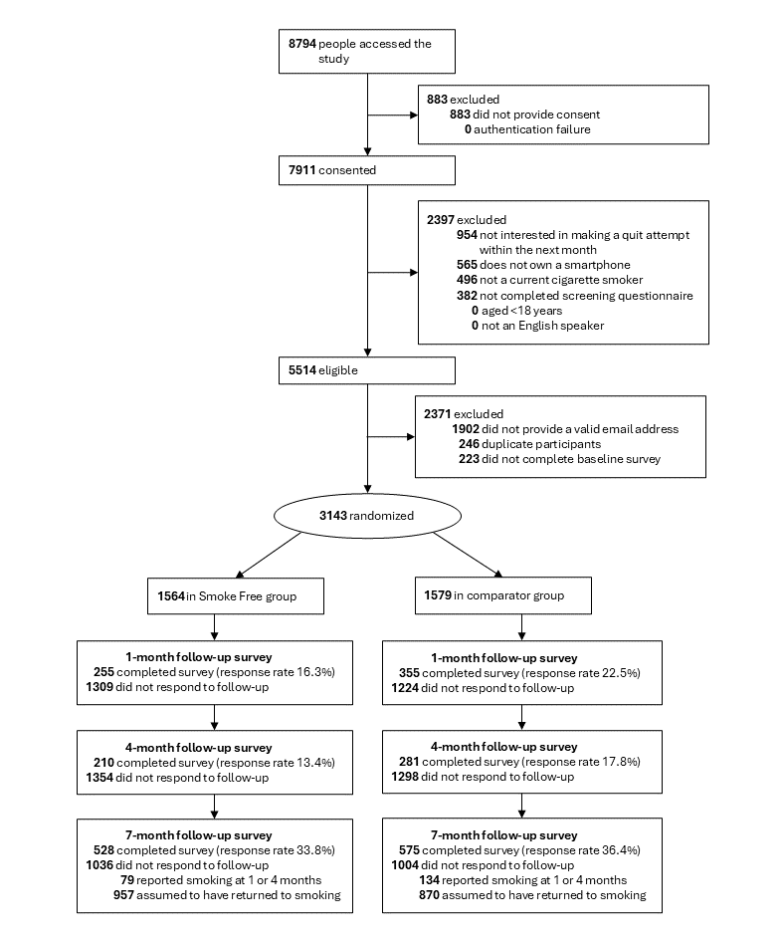
CONSORT (Consolidated Standards of Reporting Trials) flowchart.

A total of 3143 eligible participants were recruited, completed the baseline assessment, and were randomized to the intervention or comparator condition. Figure 1 shows the numbers allocated to each group and followed-up. Baseline sociodemographic and smoking characteristics were similar across groups (Multimedia Appendix 4), consistent with successful randomization.

Response rates to the 1-month, 4-month, and 7-month follow-ups were 19.4%, 15.6%, and 35.1%, respectively; 16.3%, 13.4%, and 33.8% in the Smoke Free group, and 22.5%, 17.8%, and 36.4% in the comparator group (Figure 1). Of the 2040 participants who did not respond directly to the final follow-up, 213 (10.4%) individuals reported smoking in a previous follow-up assessment, which would have classified them as smokers by our continuous abstinence primary outcome, meaning the effective follow-up rate for the primary outcome was 41.9% (2040 – 213 = 1827; 3143 – 1827 = 1316; 1316/3143); 38.8% in the Smoke Free group and 44.9% in the comparator group.

### Primary Outcome

Table 1 shows results for the primary outcome of 6-month continuous abstinence, assessed at 7-month follow-up, using an ITT analysis approach. Overall, 6.9% (218/3143) of participants reported 6-month continuous abstinence. The rate of smoking cessation was similar between participants in the Smoke Free and comparator groups (6.8% vs 7.0%, respectively). The Bayes factor favored no effect. Moderation analyses showed no significant difference in treatment effect by any characteristic (Multimedia Appendix 3).

**Table 1:** Log-binomial regression analyses of treatment effect on primary and secondary outcomes^a^.

		Comparator	Offered Smoke Free app			
		n/N (%)	n/N (%)	RR^b^ (95% CI)	*P* value	BF^c,d^
**Primary outcome**						
	6-month continuous abstinence^e^	111/1579 (7.03)	107/1564 (6.84)	0.97 (0.75 to 1.26)	.84	0.26
**Secondary outcomes**						
	Making at least one quit attempt^f^	242/1579 (15.33)	180/1564 (11.51)	0.75 (0.63 to 0.90)	.002	0.05
	3-month continuous abstinence^g^	54/1579 (3.42)	45/1564 (2.88)	0.84 (0.57 to 1.24)	.38	0.26
	6-month continuous abstinence among those who tried to quit^e,h^	48/242 (19.8)	43/180 (23.9)	1.20 (0.83 to 1.73)	.32	1.01
	Reported downloading or using the Smoke Free app at least once^e^	51/1579 (3.23)	85/1564 (5.43)	1.68 (1.20 to 2.38)	.003	28.6

^a^Intention-to-treat analysis.

^b^RR: relative risk (reference group: comparator).

^c^BF: Bayes factor.

^d^Bayes factors ≥3 can be interpreted as evidence for the alternative hypothesis, ≤1/3 as evidence for the null hypothesis, and Bayes factors between 1/3 and 3 suggest the data are insensitive to distinguishing the alternative hypothesis from the null [26,27].

^e^Assessed at 7-month follow-up.

^f^Assessed at 1-month follow-up.

^g^Assessed at 4-month follow-up.

^h^Tried to quit: reported making at least one quit attempt at 1-month follow-up.

### Secondary Outcomes

Table 1 also summarizes results relating to the secondary outcomes. Participants in the Smoke Free group had a 25% lower risk of reporting a quit attempt compared with those in the comparator group. There was no statistically significant difference in 3-month continuous abstinence rates between groups or 6-month continuous abstinence among those who tried to quit (Bayes factors for these outcomes favored no effect). Those in the Smoke Free group were 68% more likely to report having downloaded or used the Smoke Free app at least once during this study’s period than those in the comparator group, although self-reported rates were very low in both groups (5.4% vs 3.2%, respectively).

### Sensitivity Analyses

Table 2 shows results for primary and secondary outcomes restricting the intervention group to those who took up the offer of the Smoke Free app. Despite just 85/1564 (5.3%) participants in the intervention group recalling, at 7-month follow-up, having downloaded or used the Smoke Free app at least once, matching email address log-ins were verified for 355/1564 (22.7%) participants in the intervention group—indicating they had (at least briefly) taken up the offer of the app after the baseline survey. This discrepancy is likely to be largely due to loss to follow-up; most of the participants either did not respond to the 7-month follow-up or only provided data on the primary outcome. Combined, 395/1564 (25.3%) participants in the intervention group either self-reported or had verified app use. Multimedia Appendix 5 compares the baseline characteristics of these intervention participants with those of the comparator group. The 6-month continuous abstinence rate among the subset of the intervention group who took up the offer of the app was 80% higher than the comparator group (12.7% vs 7.0%; Table 2). After adjustment for baseline covariates, the difference between groups attenuated to 60% but remained statistically significant (*P*<.05; Table 2). Among this subset of the intervention group, the rate of quit attempts was 40% higher than the comparator group (21.5% vs 15.3%), 3-month continuous abstinence was 85% higher (6.3% vs 3.4%), and 6-month continuous abstinence among those who made a quit attempt was 60% higher (30.3% vs 19.8%); these differences remained statistically significant after adjustment for covariates (*P*<.05; Table 2).

**Table 2:** Sensitivity analysis: restricting the intervention group to those who took up the offer of the app (self-reported or verified)^a^.

			Comparator		Offered Smoke Free app		Unadjusted							Adjusted for baseline characteristics^b^				
			n/N (%)		n/N (%)		RR^c^ (95% CI)		*P* value		BF^d,e^		RR (95% CI)			*P* value		BF^e^
***Primary outcome***																		
	6-month continuous abstinence^f^	111/1579 (7.03)		50/395 (12.7)		1.80 (1.30-2.45)		<.001		>100		1.60 (1.16-2.18)			.003		34.1	
***Secondary outcomes***																		
	Making at least one quit attempt^g^	242/1579 (15.33)		85/395 (21.5)		1.40 (1.12-1.74)		.003		38.1		1.33 (1.06-1.64)			.01		5.28	
	3-month continuous abstinence^h^	54/1579 (3.42)		25/395 (6.3)		1.85 (1.15-2.90)		.009		14.9		1.62 (1.01-2.53)			.04		5.25	
	6-month continuous abstinence among those who tried to quit^f,i^	48/242 (19.8)		27/85 (32)		1.60 (1.05-2.37)		.02		7.94		1.48^j^ (1.00-2.21)			.05		3.73	

^a^Intention-to-treat analysis.

^b^Adjusted for age, financial status, level of addiction (time to first cigarette after waking), and current use of evidence-based support (nicotine replacement therapy on prescription, varenicline, bupropion, face-to-face support, or e-cigarettes).

^c^RR: relative risk (reference group: comparator).

^d^BF: Bayes factor.

^e^Bayes factors ≥3 can be interpreted as evidence for the alternative hypothesis, ≤1/3 as evidence for the null hypothesis, and Bayes factors between 1/3 and 3 suggest the data are insensitive to distinguish the alternative hypothesis from the null [26,27].

^f^Assessed at 7-month follow-up.

^g^Assessed at 1-month follow-up.

^h^Assessed at 4-month follow-up.

^i^Tried to quit: reported making at least one quit attempt at 1-month follow-up.

^j^There were convergence issues for this model. To ensure convergence: (1) iterations were increased to 1000 and the expectation-maximization algorithm was used rather than the default iteratively reweighted least squares algorithm [28], and (2) evidence-based support was excluded as a covariate.

Table 3 shows results for primary and secondary outcomes with analyses rerun (1) on complete cases and (2) with missing data imputed using multiple imputation. In both analyses, there was no statistically significant difference between groups on quitting outcomes (Bayes factors indicated the data were insensitive or favored no effect). Participants in the intervention group were significantly more likely to report having downloaded or used the Smoke Free app at least once.

**Table 3:** Sensitivity analyses: complete cases and multiple imputation.

				Comparator		Offered Smoke Free app						
				n/N (%)		n/N (%)		RR^a^ (95% CI)		*P* value		BF^b,c^
**Complete cases**												
	*Primary outcome*											
		6-month continuous abstinence^d^	111/712 (15.6)		107/609 (17.6)		1.13 (0.88-1.44)		.33		0.75	
	*Secondary outcomes*											
		Making at least one quit attempt^e^	242/354 (68.4)		180/255 (70.6)		1.03 (0.93-1.15)		.56		0.22	
		3-month continuous abstinence^f^	54/438 (12.3)		45/309 (14.6)		1.18 (0.81-1.70)		.38		0.91	
		6-month continuous abstinence among those who tried to quit^d,g^	48/225 (21.3)		43/160 (26.9)		1.26 (0.88-1.80)		.21		1.41	
		Reported downloading or using the Smoke Free app at least once^d^	51/212 (24.1)		85/176 (48.3)		2.01 (1.52-2.69)		<.001		>100	
**Multiple imputation**												
	*Primary outcome*											
		6-month continuous abstinence^d^	20.01 (316/1579)		22.38 (350/1564)		1.12 (0.94-1.33)		.21		0.84	
	*Secondary outcomes*											
		Making at least one quit attempt^e^	63.46 (1002/1579)		61.32 (959/1564)		0.97 (0.87-1.07)		.49		0.08	
		3-month continuous abstinence^f^	17.04 (269/1579)		19.12 (299/1564)		1.12 (0.91-1.39)		.29		0.72	
		6-month continuous abstinence among those who tried to quit^d,g^	23.58 (241/1022)		26.5 (254/959)		1.22 (0.85-1.74)		.28		1.14	
		Reported downloading or using the Smoke Free app at least once^d^	26.35 (416/1579)		45.27 (708/1564)		1.72 (1.39-2.13)		<.001		>100	

^a^RR: relative risk (reference group: comparator).

^b^BF: Bayes factor.

^c^Bayes factors ≥3 can be interpreted as evidence for the alternative hypothesis, ≤1/3 as evidence for the null hypothesis, and Bayes factors between 1/3 and 3 suggest the data are insensitive to distinguish the alternative hypothesis from the null [26,27].

^d^Assessed at 7-month follow-up.

^e^Assessed at 1-month follow-up.

^f^Assessed at 4-month follow-up.

^g^Tried to quit: reported making at least one quit attempt at 1-month follow-up.

Just 108 participants reported their resting heart rate at baseline and 7-month follow-up. When we defined successful quits as self-reported abstinence plus a reduction in mean resting heart rate of ≥5 beats per minute, the rate of 6-month continuous abstinence did not differ significantly between intervention and comparator arms (5/1564, 0.32% vs 9/1579, 0.57%; RR 0.56, 95% CI 0.17-1.62, *P*=.30). The Bayes factor indicated the data were insensitive (Bayes factor=0.51).

Assuming different rates of abstinence among participants who were lost to follow-up had little effect on our primary outcome (Multimedia Appendix 6).

## Discussion

Among motivated smokers provided with very brief advice to quit, there was no significant difference in 6-month quit rates between participants randomized to receive the offer of the Smoke Free app plus follow-up and those randomized to follow-up only. This result was observed across analyses using ITT analysis, complete cases, and multiple imputation, and there were similar results on the secondary outcomes. However, when the intervention group was restricted to those who took up the offer of the Smoke Free app a significant benefit of treatment was observed, with participants in the intervention group being 80% more likely to report abstinence than those in the comparator group on the primary outcome, with similar results on secondary outcomes. This was only partly explained by differences in baseline characteristics, with the effect remaining at 60% (a statistically significant difference) after adjustment for age, financial status, level of addiction, and current use of evidence-based cessation support.

There was no significant difference in 6-month continuous abstinence among those who tried to quit in ITT analysis, complete case, and multiply imputed analyses. These analyses were limited by the low response to the 1-month follow-up survey (which assessed quit attempts) and Bayes factors indicated the data were insensitive. The data showed a benefit of treatment when the intervention group was restricted to those who took up the offer of the Smoke Free app, with the intervention group 60% more likely to report abstinence than those in the comparator group.

ITT analyses indicated a lower rate of quit attempts in the first 4 weeks in the intervention group compared with the comparator. However, this was not consistently observed across sensitivity analyses that used complete cases or multiple imputation, which showed no significant effect (with Bayes factors favoring no difference). It is likely the ITT analysis result was an artifact resulting from the lower response rate to the 1-month follow-up survey in the intervention versus comparator group (16% vs 23%, meaning a greater proportion of the intervention group were assumed not to have made a quit attempt) rather than any genuine difference between the groups. Indeed, when the intervention group was restricted to those who took up the offer of the Smoke Free app, a significant benefit of treatment was observed, with participants in the intervention group 40% more likely to report attempting to quit.

There was no statistically significant difference in 3-month continuous abstinence rates between groups. Analyses of this outcome were limited by the low response rate to the 4-month follow-up survey (16%). Bayes factors indicated the data were insensitive for all analyses except the ITT analysis, which favored no effect, and the analysis restricting the intervention group to those who took up the offer of the app, which showed a significant benefit of treatment (85% more likely to report 3-month continuous abstinence).

Participants in the intervention group were significantly more likely to report having downloaded or used the Smoke Free app at least once during this study’s period, although uptake of the offer of the app was low across self-report (5%) and validated (23%) measures (25% overall). Prevalence of self-reported uptake was suppressed by the low response to the final follow-up survey, particularly because many responders (ie, those who responded via email or telephone) only provided data on the primary outcome. However, the validated measure of treatment uptake will have captured most of the participants who took up the offer of the app, as long as they used the same email address to sign up for this study and register for the app.

To our knowledge, this is the first study to test the effectiveness of a smoking cessation app compared with unaided quitting. It differs from other large trials of smoking cessation apps [9] not only in its comparator group (ie, follow-up only rather than active treatment) but also in the way it was advertised, making no reference to smartphone apps until participants were enrolled. It did not aim to target smokers interested in quitting with the support of an app, but rather any smoker motivated to make a quit attempt. Thus, the relatively low uptake of the offer of the app in the intervention group is not surprising. Representative observational data show low rates of adoption of digital aids for smoking cessation, with fewer than 3% of smokers who have tried to quit reporting using a digital cessation aid (app or website) [29]. Our results suggest that while not every smoker will be interested in trying them, the use of smoking cessation apps can be increased by directing smokers to this type of support (395/1564 [25.26%] of those offered registered an account with the app). Our data also suggest that the Smoke Free app boosted quit rates among smokers who used it. However, it is possible that this may have reflected participants who downloaded the app as being more serious and motivated about quitting (as indicated by acting and downloading or using the app) and that other cessation aids could have produced similar results (vs no aid). Given the wide reach of smartphone apps, it is possible that initiatives to increase smokers’ awareness of smoking cessation apps could have a meaningful impact on rates of cessation at the population level even if only a minority of smokers take up the use of app-based support (even a small percentage of a very large number can be a large number). Analyses based on complete cases and multiple imputed data indicated offering the app increased the risk of 6-month continuous abstinence by 10% versus follow-up only. While this would generally be considered a small effect size for a behavioral intervention, small effects of treatments that aid smoking cessation can be clinically significant because of the very large health gains that accrue from stopping smoking [30]. Moreover, offering the app to all smokers at a population level (a low-cost, highly scalable intervention) could result in a large number of successful quits. Future trials should prioritize establishing the relative effectiveness and cost-effectiveness of apps compared with other evidence-based smoking cessation treatments.

Strengths of this study include the large sample size, the wide geographic scope, and the 6-month follow-up duration [22]. The pragmatic design offers real-world insights, focusing on the offer rather than the use of the app, as not every smoker will want to use digital support or apps. There were also several limitations. First, there was a high rate of attrition. Given the pragmatic design and light-touch intervention, we anticipated that response to follow-up attempts would be relatively low [14] and concentrated our limited resources on maximizing response to the final follow-up at 7 months. Second, our study should have been powered for a smaller effect size given the low rate of uptake in the intervention group. With just 25.26% (395/1564) of participants taking up the offer of the app and an observed effect size of RR ~1.3-1.5 among this group, future trials of this nature would need to be powered to detect smaller effects (RR ~1.1), requiring samples in the region of 64,000 participants. This is not unfeasible given the numbers involved in some digital trials [31]. Future studies could investigate barriers to app use to explore the low rate of uptake of the offer of free access to a paid app in a motivated group of smokers. Third, we did not undertake remote biochemical data collection to verify abstinence. While biochemical verification is widely considered the gold standard for evaluating cessation outcomes [22,32], the Society for Research on Nicotine and Tobacco Subcommittee on Biochemical Verification has advised that in large-scale population-based trials such as this one, where face-to-face contact is limited and data are optimally collected online, the added precision gained by biochemical verification may be offset by methodological problems in such a way that its use is not required and may not be desirable [32,33]. Given our study’s large, geographically dispersed participant sample, collecting biological samples or conducting remote observation of rapid tests would have been costly and logistically challenging. It may also have reduced the representativeness of the sample (if smokers unwilling to provide samples were ineligible to participate) or increased the rate of missing outcome data (if logistical complexity and participant burden reduced the likelihood of follow-up response or resulted in unusable samples) [34]. We explored the possibility of verifying abstinence via a reduction in self-reported resting heart rate, but only a small minority of participants provided this data and the analysis was insensitive. Collecting this data was not an important aspect of our follow-up strategy and it may be feasible in future trials with additional incentive. Finally, while we recruited an international sample, with no restrictions on location, the majority (97.26%, 3057/3143) of participants were from 5 high-income Western countries, which may limit generalizability. Further investigation is required in low- and middle-income countries, where the potential benefits of smoking cessation apps may be greater in the absence of comprehensive and affordable cessation support.

In conclusion, among motivated smokers provided with very brief advice to quit, the Smoke Free app did not have a detectable benefit for cessation compared with follow-up only. However, the app increased quit rates among smokers who were randomized to receive it and who downloaded it.

## Appendix

Multimedia Appendix 1 Additional information on methods.

Multimedia Appendix 2 Amendments to the analysis plan.

Multimedia Appendix 3 Results of moderation analyses.

Multimedia Appendix 4 Baseline characteristics.

Multimedia Appendix 5 Baseline characteristics, restricting intervention group to those who took up the offer of the app.

Multimedia Appendix 6 Impact of assuming different rates of abstinence in participants lost to follow-up.

Multimedia Appendix 7 CONSORT 2010 checklist. CONSORT: Consolidated Standards of Reporting Trials.

## Abbreviations

ITT: intention-to-treat analysis
ISRCTN: International Standard Randomised Controlled Trial Number
RCT: randomized controlled trial
RR: relative risk

## Data Availability

Data are available on the Open Science Framework [15].
