# Supplementary material for: Effectiveness of the Offer of the Smoke Free Smartphone App Compared With No Intervention for Smoking Cessation: Pragmatic Randomized Controlled Trial
Source: J Med Internet Res. 2024 Nov 15;26:e50963. doi: 10.2196/50963 (PMC11607577; doi:10.2196/50963)
Supplement: Multimedia Appendix 1 [file jmir_v26i1e50963_app1.pdf]

Additional information on methods

Contents:

- Study timeline
- Recruitment advert posted on Facebook and Twitter
- Message shown to both intervention and comparator groups at the end of the baseline survey
- More detail on the *Smoke Free* app

Study timeline

Month	**0**	**1**	**4**	**7**
	- Recruitment<br>- Consent<br>- Screening<br>- Baseline questionnaire<br>- Randomisation<br>- Brief message encouraging making a quit attempt	- 1-month follow-up questionnaire	- 4-month follow-up questionnaire	- 7-month follow-up questionnaire

Recruitment advert posted on Facebook and Twitter

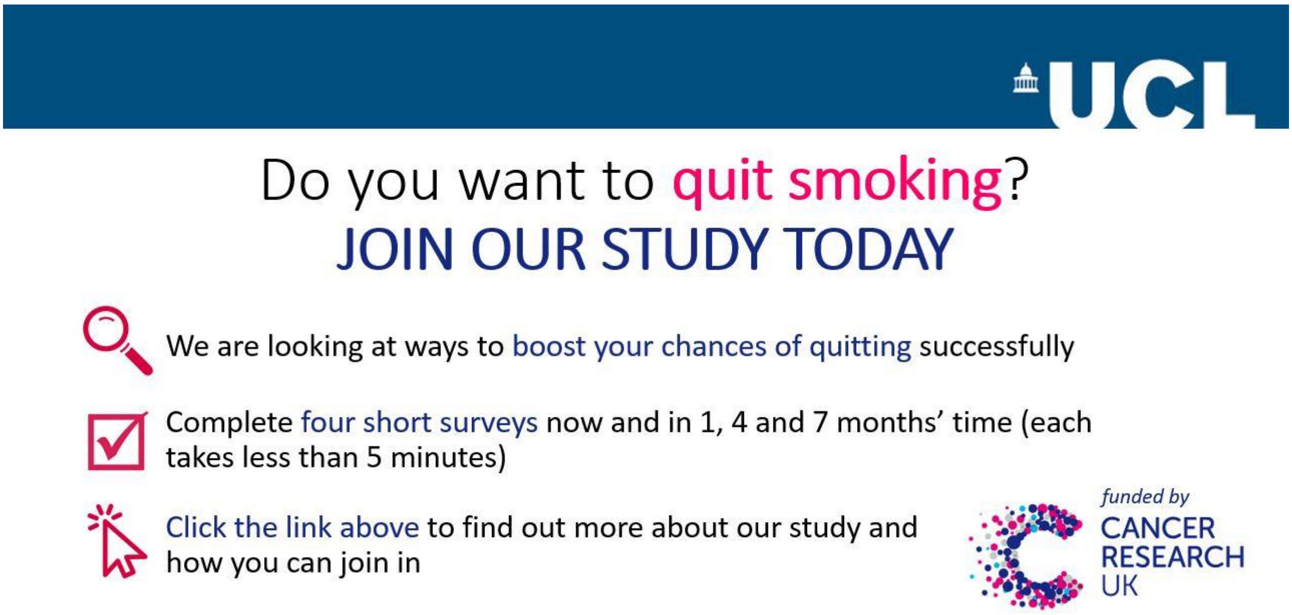

Message shown to comparator group at the end of the baseline survey.

You said you wanted to make a quit attempt in the next 4 weeks. That's great! Quitting is the single best thing you can do for your health. When will you start? Think carefully about the best date and time for you. It might be right now.

The first 24 hours are crucial - get past those and you'll be twice as likely to stay off cigarettes for good! One thing that we know helps is making a serious commitment to being a non-smoker. Keeping track of your progress is another good technique.

Thank you again for completing the survey and taking part in our study. We'll send you an email in a few weeks to ask how you're getting on. Please reply, it makes a big difference to our Cancer Research UK funded study if you do.

Message shown to intervention group at the end of the baseline survey.

You said you wanted to make a quit attempt in the next 4 weeks. That's great! Quitting is the single best thing you can do for your health. When will you start? Think carefully about the best date and time for you. It might be right now.

The first 24 hours are crucial - get past those and you'll be twice as likely to stay off cigarettes for good! One thing that we know helps is making a serious commitment to being a non-smoker. Keeping track of your progress is another good technique.

But perhaps the best thing you can do is get support. Stopping smoking isn't easy, so getting help is smart. You're much more likely to be successful with support.

There's lots more support in Smoke Free now, and you can access it all for free. The two big new additions are an automated quit coach that takes you through a 100-day programme and is proven to help. There are also real-life stop smoking experts on-hand 18 hours a day to guide and support you through all steps of your quitting journey.

To reiterate – this is all completely free.

[DOWNLOAD NOW – link]

Thank you again for completing the survey and taking part in our study. We'll send you an email in a few weeks to ask how you're getting on. Please reply, it makes a big difference to our Cancer Research UK funded study if you do.

More detail on the *Smoke Free* app

The app has several components:

1. A calculator which tracks the total amount of money not spent on buying cigarettes and the number of cigarettes not smoked ('Dashboard');
2. A calendar which tracks the amount of time elapsed since cessation ('Dashboard');
3. A scoreboard which awards virtual 'badges' to users for not smoking ('Badges');
4. Progress indicators which inform users of the health improvements made since the start of their quit attempt (e.g. pulse rate, oxygen levels, carbon monoxide levels; 'Dashboard');
5. A diary which tracks the frequency, strength, location and triggers of cravings to smoke ('Diary');
6. A graph which displays the frequency, location, strength and triggers of cravings to smoke ('Cravings');
7. Daily missions which are assigned from the start of a user's quit date for one calendar month ('Missions');
8. A chatbot which delivers evidence-based guidance about quitting smoking via a conversational interface which resembles text messaging;
9. 24/7 access to National Centre for Smoking Cessation and Training (NCSCT)-trained advisors;
10. Advisor-led stop smoking clinics held in-app four times a day.

Behaviour change techniques included in the app are summarised in the trial protocol: https://onlinelibrary.wiley.com/doi/abs/10.1111/add.14652
