# Supplementary material for: Effectiveness of the Offer of the Smoke Free Smartphone App Compared With No Intervention for Smoking Cessation: Pragmatic Randomized Controlled Trial
Source: J Med Internet Res. 2024 Nov 15;26:e50963. doi: 10.2196/50963 (PMC11607577; doi:10.2196/50963)
Supplement: Multimedia Appendix 3 [file jmir_v26i1e50963_app3.pdf]

Results of moderation analyses

Table S1 summarises tests of moderation of treatment effects by baseline characteristics (gender, level of addiction, age, education, financial situation, and previous experience with a smoking cessation app). For the primary outcome of 6-month continuous abstinence, the treatment effect did not differ significantly according to any characteristic. However, there was some evidence of moderation of secondary outcomes.

For making a quit attempt, there was a significant interaction between group and level of cigarette addiction (**Table S1**; **Figure S1**). Stratified analyses indicated that the likelihood of making a quit attempt was only significantly lower in the *Smoke Free* group versus comparator group among participants who were more addicted (i.e. those who reported smoking their first cigarette of the day within 5 minutes of waking: RR 0.58, 95% CI 0.42-0.78, *p*<0.001, or 6-30 minutes after waking: RR 0.73, 95% CI 0.55-0.97, *p*=0.033). There was no significant group difference in quit attempts among those who smoked their first cigarette 31-60 minutes (RR 0.92, 95% CI 0.55-1.54, *p*=0.737) or >60 minutes after waking (RR 1.31, 95% CI 0.80-2.18, *p*=0.288).

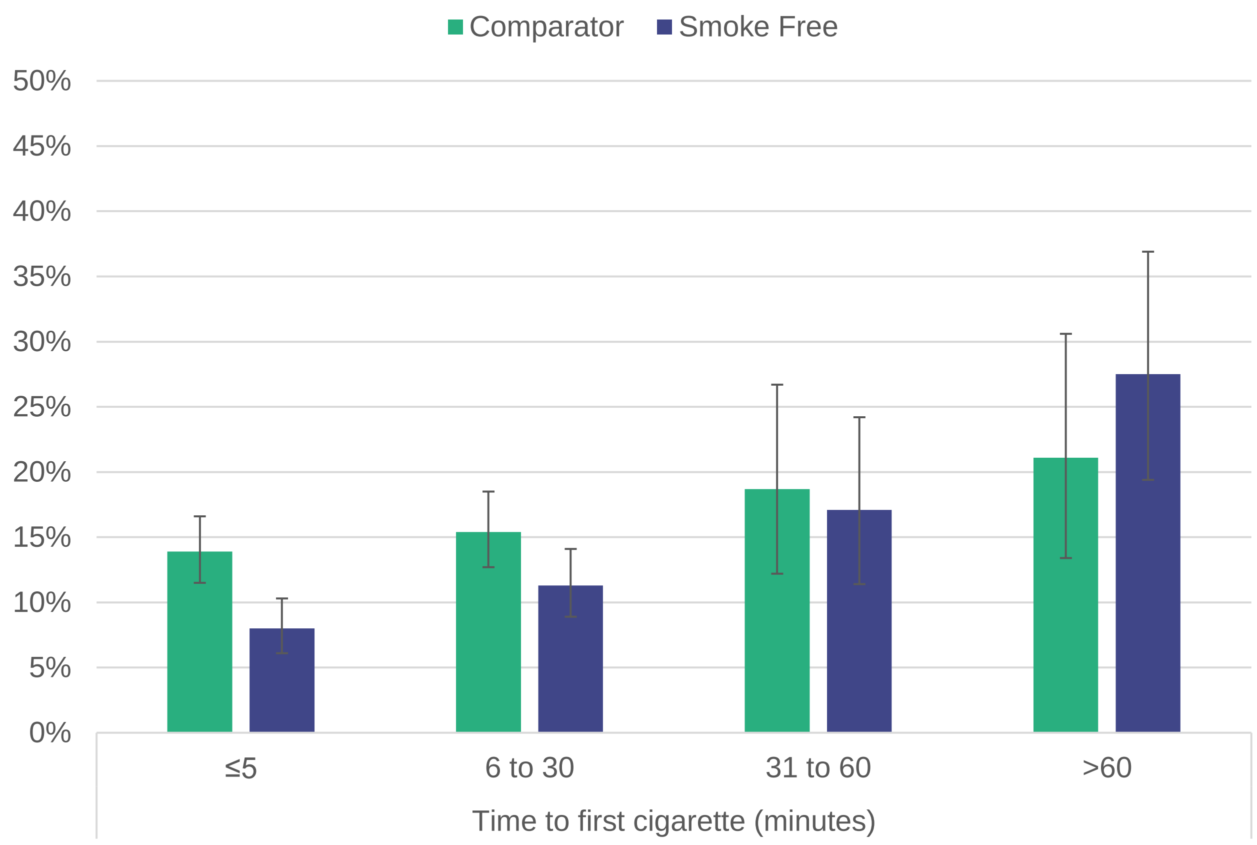

Figure S1. Rates of quit attempts by level of addiction (indexed by time to first cigarette after waking) and group

Table S1. Tests of moderation of treatment effect by key baseline characteristics

	Group*gender			Group*level of addiction			Group*age		
	RR	95% CI	*p*	RR	95% CI	*p*	RR	95% CI	*p*
Primary outcome									
6-month continuous abstinence[1]	1.03	0.57 to 1.86	0.910	0.80	0.62 to 1.03	0.090	1.21	0.59 to 2.49	0.603
Secondary outcomes									
Making at least one quit attempt[2]	0.84	0.57 to 1.23	0.369	**1.31**	**1.10 to 1.57**	**0.003**	0.64	0.41 to 1.01	0.054
3-month continuous abstinence[3]	1.18	0.52 to 2.70	0.692	0.89	0.60 to 1.30	0.537	0.81	0.31 to 2.13	0.665
6-month continuous abstinence among those who tried to quit[1,4]	1.11	0.50 to 2.45	0.803	0.87	0.63 to 1.22	0.420	1.21	0.49 to 2.93	0.678
Reported downloading or using the Smoke Free app at least once[1]	1.07	0.50 to 2.24	0.861	0.96	0.69 to 1.34	0.809	**2.37**	**1.09 to 5.22**	**0.029**
	Group*education			Group*financial situation			Group*previous experience with a smoking cessation app		
	RR	95% CI	*p*	RR	95% CI	*p*	RR	95% CI	*p*
Primary outcome									
6-month continuous abstinence[1]	0.47	0.18 to 1.16	0.112	1.02	0.75 to 1.40	0.894	0.74	0.44 to 1.23	0.246
Secondary outcomes									
Making at least one quit attempt[2]	1.63	0.83 to 3.44	0.170	0.93	0.75 to 1.16	0.536	0.85	0.94 to 1.50	0.369
3-month continuous abstinence[3]	0.80	0.14 to 4.43	0.784	0.97	0.59 to 1.58	0.890	0.76	0.35 to 1.66	0.489
6-month continuous abstinence among those who tried to quit[1,4]	**0.20**	**0.03 to 0.79**	**0.036**	1.24	0.80 to 1.92	0.337	0.61	0.29 to 1.28	0.195
Reported downloading or using the Smoke Free app at least once[1]	1.22	0.24 to 5.67	0.795	0.90	0.59 to 1.36	0.607	1.07	0.50 to 2.24	0.861

[1]Assessed at 7-month follow-up.
[2]Assessed at 1-month follow-up.
[3]Assessed at 4-month follow-up.
[4]Tried to quit = reported making at least one quit attempt at 1-month follow-up.
Note: intention-to-treat analysis with missing-equals-smoking imputation. Independent variables were coded as follows: group (0=comparator, 1=Smoke Free), gender (0=male, 1=female), level of addiction (time to first cigarette: 0=within 5 minutes, 1=6-30 minutes, 2=31-60 minutes, 4=after 60 minutes), age (0=<35, 1=≥35), education (0=no post-16 qualifications, 1=post-16 qualifications), financial situation (0=don't meet basic expenses, 1=just meet basic expenses, 2=meets needs with a little left, 3=live comfortably), previous experience with a smoking cessation app (0=no, 1=yes). Bold font indicates statistically significant interactions.

For 6-month continuous abstinence among those who tried to quit, there was a significant interaction between group and education (**Table S1**, **Figure S2**). While the risk of abstinence did not differ significantly by group at either level of education, the point estimate was lower among those with post-16 qualifications (RR 1.07, 95% CI 0.73-1.56, *p*=0.743) than those without (RR 5.45, 95% CI 1.37-36.73, *p*=0.036).

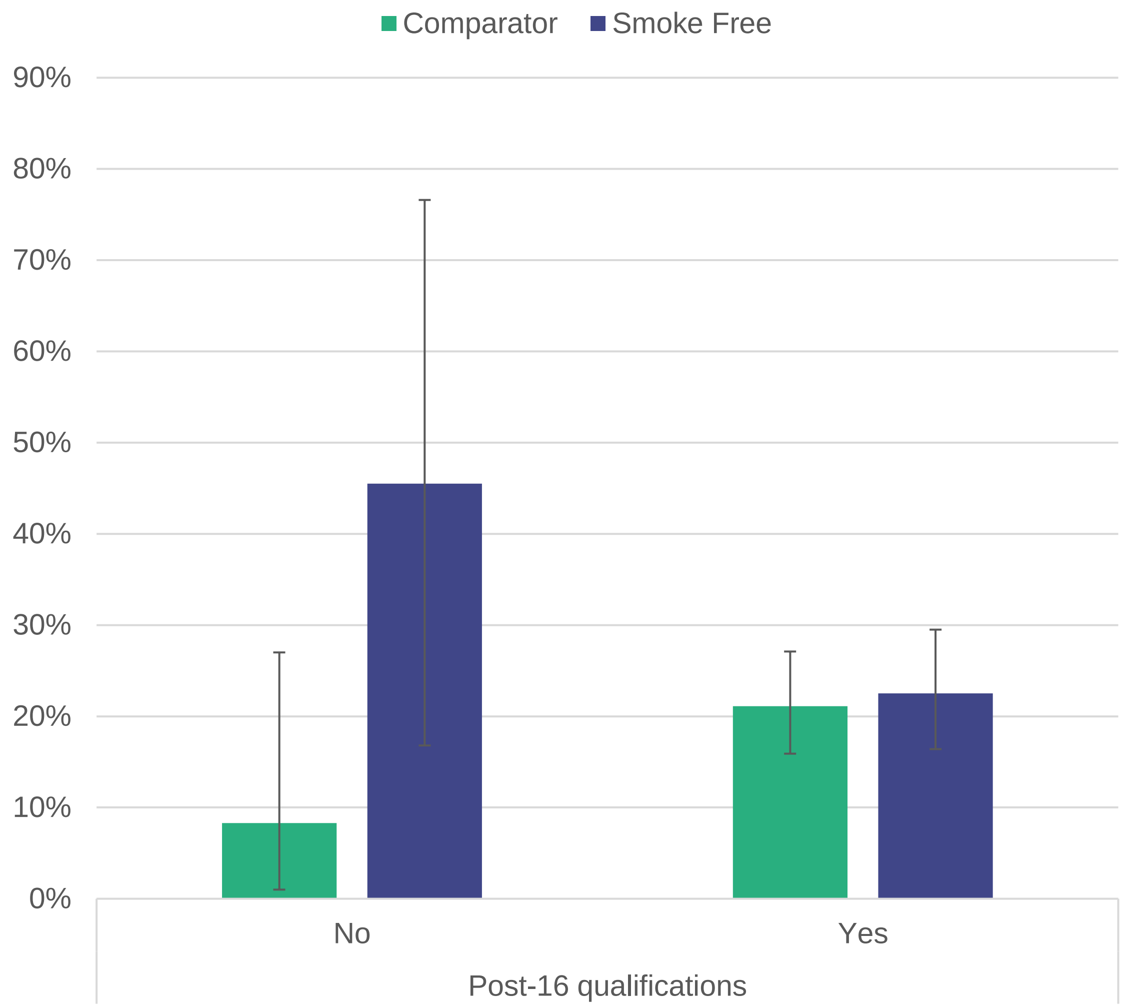

Figure S2. Rates of 6-month continuous abstinence among those who tried to quit by level of education and group

For downloading or using the *Smoke Free* app at least once during the study period, there was a significant interaction between group and age (**Table S1**; **Figure S3**). The risk of app use was significantly higher in the *Smoke Free* group versus comparator group among older participants (≥35 years: RR 2.08, 95% CI 1.40-3.15, *p*<0.001) but did not differ significantly by group among those aged <35 (RR 0.88, 95% CI 0.45-1.71, *p*=0.699).

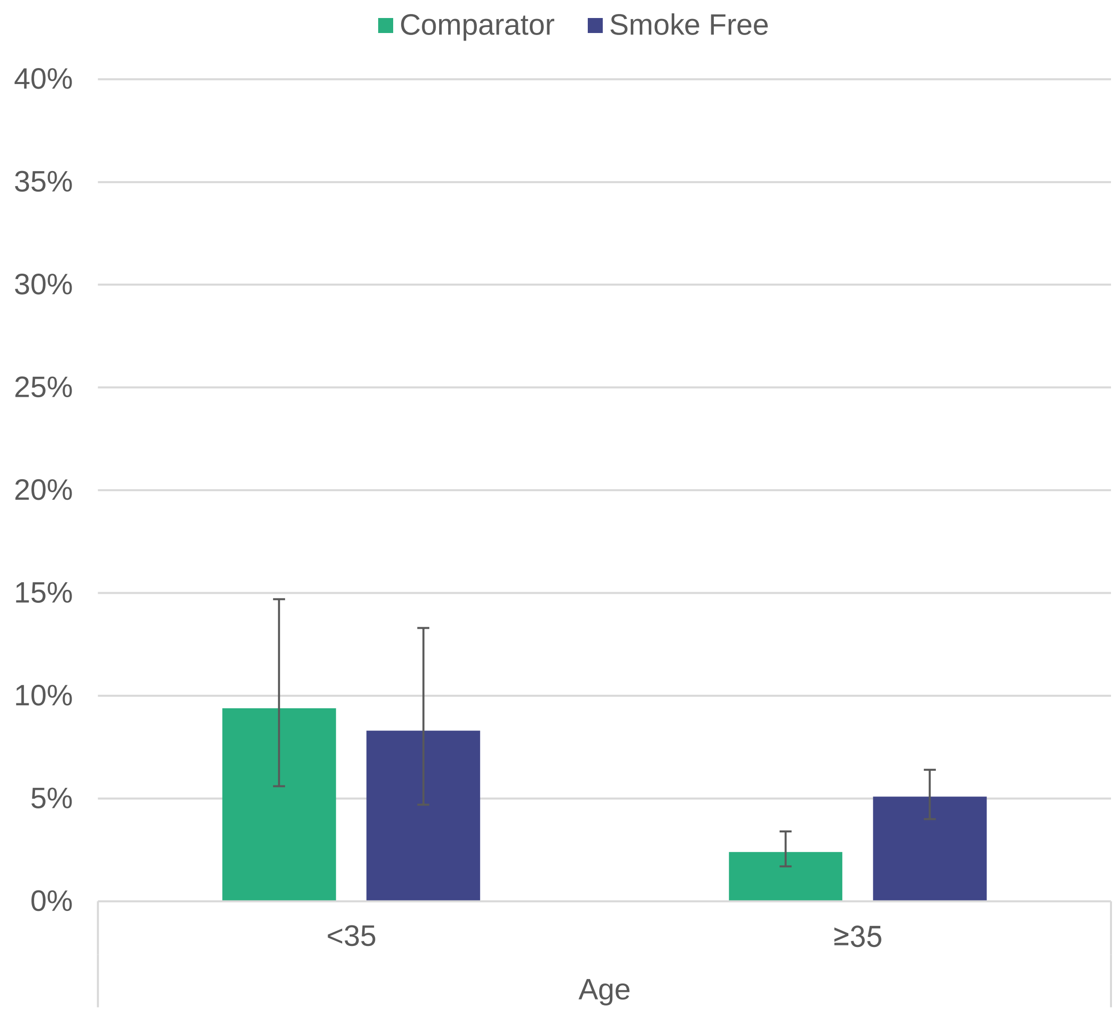

Figure S3. Rates of reporting downloading or using the Smoke Free app at least once by age and group
