# Supplementary material for: Effectiveness of the Offer of the Smoke Free Smartphone App Compared With No Intervention for Smoking Cessation: Pragmatic Randomized Controlled Trial
Source: J Med Internet Res. 2024 Nov 15;26:e50963. doi: 10.2196/50963 (PMC11607577; doi:10.2196/50963)
Supplement: Multimedia Appendix 4 [file jmir_v26i1e50963_app4.pdf]

Baseline characteristics

Table S1. Baseline characteristics of participants randomised to each condition

	Total (n=3143)		Comparator (n=1579)		Offered *Smoke Free* app (n=1564)	
	%	n	%	n	%	n
Age (years)						
18-34	11.5	361	11.4	180	11.6	181
35-64	81.5	2562	82.0	1294	81.1	1268
65+	7.0	220	6.7	105	7.4	115
Gender						
Male	25.0	785	24.2	382	25.8	403
Female	74.7	2349	75.5	1192	74.0	1157
Other	0.3	9	0.3	5	0.3	4
Post-16 qualifications	90.5	2845	90.4	1427	90.7	1418
Financial status						
Live comfortably	5.6	176	5.9	93	5.3	83
Meet needs with a little left	32.8	1031	33.5	529	32.1	502
Just meet basic expenses	41.6	1307	39.7	627	43.5	680
Don’t meet basic expenses	20.0	629	20.9	330	19.1	299
Country of residence						
UK	45.4	1426	45.8	723	45.0	703
USA	33.9	1065	34.3	542	33.4	523
Canada	8.6	270	8.0	127	9.1	143
Ireland	6.4	201	5.8	91	7.0	110
Australia	3.0	95	3.0	48	3.0	47
Other	2.7	86	3.0	48	2.4	38
English as first language	95.4	2997	95.8	1513	94.9	1484
Time to first cigarette						
≤5 minutes	45.3	1424	46.0	726	44.6	698
6-30 minutes	39.6	1246	40.2	635	39.1	611
31-60 minutes	8.6	269	7.8	123	9.3	146
>60 minutes	6.5	204	6.0	95	7.0	109
History of serious quit attempts						
Never	7.1	223	7.3	115	6.9	108
Yes – not in the past year	58.9	1852	59.6	941	58.3	911
Yes – in the past year	34.0	1068	33.1	523	34.9	545

Table continued on next page.

Table S1. (continued)

	Total (n=3143)		Comparator (n=1579)		Offered *Smoke Free* app (n=1564)	
	%	n	%	n	%	n
Past use of cessation support						
Prescription NRT	52.4	1648	53.1	839	51.7	809
NRT bought over the counter	29.6	930	30.0	474	29.2	456
Varenicline	16.5	518	15.6	246	17.4	272
Bupropion	14.6	460	14.1	222	15.2	238
Face-to-face behavioural support	8.2	257	8.7	138	7.6	119
Telephone support	6.2	195	5.9	93	6.5	102
Written self-help materials	23.9	751	24.3	384	23.5	367
Websites	10.6	333	11.0	173	10.2	160
Apps	48.7	1531	47.7	753	49.7	778
E-cigarette or other vaping device	16.4	516	17.0	269	15.8	247
Other	3.3	102	2.7	43	3.8	59
None of the above	12.8	402	13.1	206	12.5	196
Current use of cessation support						
Prescription NRT	2.5	78	2.5	39	2.5	39
NRT bought over the counter	9.7	304	9.1	143	10.3	161
Varenicline	1.9	58	2.0	31	1.7	27
Bupropion	0.7	21	0.7	11	0.6	10
Face-to-face behavioural support	0.2	7	0.1	2	0.3	5
Telephone support	0.8	25	0.4	7	1.2	18
Written self-help materials	1.8	57	1.8	28	1.9	29
Websites	2.7	85	2.4	38	3.0	47
Apps	3.9	123	4.0	63	3.8	60
E-cigarette or other vaping device	12.2	382	12.3	194	12.0	188
Other	0.9	28	0.8	12	1.0	16
None of the above	69.8	2195	70.6	1114	69.1	1081
	Mean	SD	Mean	SD	Mean	SD
Age (years)	49.0	11.5	48.9	11.4	49.2	11.6
Cigarettes per day	18.1	9.4	18.2	10.0	18.0	8.7
Resting heart rate*	75.2	18.2	75.4	18.7	74.9	17.6

NRT, nicotine replacement therapy. SD, standard deviation.

* If participants had a heart monitoring device (e.g. Fitbit, Apple watch); this was not a required field (missing overall 72.73%, n=2286; *Smoke Free* 71.82% n=1134; comparator 73.66%, n=1152).
