# Supplementary material for: Effectiveness of the Offer of the Smoke Free Smartphone App Compared With No Intervention for Smoking Cessation: Pragmatic Randomized Controlled Trial
Source: J Med Internet Res. 2024 Nov 15;26:e50963. doi: 10.2196/50963 (PMC11607577; doi:10.2196/50963)
Supplement: Multimedia Appendix 5 [file jmir_v26i1e50963_app5.pdf]

Baseline characteristics, restricting intervention group to those who took up the offer of the app

Table S1. Baseline characteristics of participants randomised to each condition restricting the intervention group to those who took up the offer of the app

	Comparator (n=1579)		Took up the offer of the *Smoke Free* app (n=395)	
	%	n	%	N
Age (years)				
18-34	11.4	180	15.4	61
35-64	82.0	1294	79.2	313
65+	6.7	105	5.3	21
Gender				
Male	24.2	382	28.9	114
Female	75.5	1192	70.6	279
Other	0.3	5	0.2	2
Post-16 qualifications	90.4	1427	95.2	376
Financial status				
Live comfortably	5.9	93	5.6	2
Meet needs with a little left	33.5	529	29.6	117
Just meet basic expenses	39.7	627	43.3	171
Don't meet basic expenses	20.9	330	21.5	85
Country of residence				
UK	45.8	723	51.1	202
USA	34.3	542	26.3	104
Canada	8.0	127	8.6	34
Ireland	5.8	91	6.8	27
Australia	3.0	48	3.8	15
Other	3.0	48	3.3	13
English as first language	95.8	1513	92.7	366
Time to first cigarette				
≤5 minutes	46.0	726	41.0	162
6-30 minutes	40.2	635	37.5	148
31-60 minutes	7.8	123	11.5	45
>60 minutes	6.0	95	10.1	40
History of serious quit attempts				
Never	7.3	115	6.6	26
Yes – not in the past year	59.6	941	56.5	223
Yes – in the past year	33.1	523	37.0	146

Table continued on next page.

Table S1. (continued)

	Comparator (n=1579)		Took up the offer of the *Smoke Free* app (n=355)	
	%	n	%	n
Past use of cessation support				
Prescription NRT	53.1	839	53.7	212
NRT bought over the counter	30.0	474	29.6	117
Varenicline	15.6	246	21.3	84
Bupropion	14.0	222	16.5	65
Face-to-face behavioural support	8.7	138	9.6	38
Telephone support	5.9	93	8.6	34
Written self-help materials	24.3	384	24.6	97
Websites	11.0	173	12.2	48
Apps	47.7	753	55.2	218
E-cigarette or other vaping device	17.0	269	24.1	95
Other	2.7	43	4.1	16
None of the above	13.1	206	12.7	50
Current use of cessation support				
Prescription NRT	2.5	39	3.0	12
NRT bought over the counter	9.1	143	8.6	34
Varenicline	2.0	31	3.0	12
Bupropion	0.7	11	0.8	3
Face-to-face behavioural support	0.1	2	0.3	1
Telephone support	0.4	7	1.3	5
Written self-help materials	1.8	28	1.5	6
Websites	2.4	38	3.8	15
Apps	4.0	63	6.3	25
E-cigarette or other vaping device	12.3	194	13.4	53
Other	0.8	12	1.0	1
None of the above	70.6	1114	65.3	258
	Mean	SD	Mean	SD
Age (years)	48.9	11.4	47.3	11.5
Cigarettes per day	18.2	10.0	17.4	8.5
Resting heart rate*	75.4	18.7	75.0	18.0

NRT, nicotine replacement therapy. SD, standard deviation.
* If participants had a heart monitoring device (e.g. Fitbit, Apple watch); this was not a required field
