# Supplementary material for: Effectiveness of the Offer of the Smoke Free Smartphone App Compared With No Intervention for Smoking Cessation: Pragmatic Randomized Controlled Trial
Source: J Med Internet Res. 2024 Nov 15;26:e50963. doi: 10.2196/50963 (PMC11607577; doi:10.2196/50963)
Supplement: Multimedia Appendix 6 [file jmir_v26i1e50963_app6.pdf]

Impact of assuming different rates of abstinence in participants lost to follow-up

Table S1. Sensitivity analysis testing the impact of assuming different rates of abstinence in those not followed-up on the primary outcome

Imputed abstinence rate in missing*	Group	Missing / N	Abstinence rate (n)†	RR† [95% CI]
0%	Comparator	870 / 1579	7.03% (111.0)	Ref
	Smoke Free	957 / 1564	6.84% (107.0)	0.97 [0.75 to 1.13]
10%	Comparator	870 / 1579	12.5% (198.0)	Ref
	Smoke Free	957 / 1564	13.0% (202.7)	1.04 [0.86 to 1.24]
20%	Comparator	870 / 1579	18.0% (285.0)	Ref
	Smoke Free	957 / 1564	19.1% (298.4)	1.06 [0.91 to 1.22]
30%	Comparator	870 / 1579	23.6% (372.0)	Ref
	Smoke Free	957 / 1564	25.2% (394.1)	1.07 [0.95 to 1.21]
40%	Comparator	870 / 1579	29.1% (459.0)	Ref
	Smoke Free	957 / 1564	31.3% (489.8)	1.08 [0.97 to 1.20]

* Imputed abstinence rate among participants who were missing at 7-month follow-up and did not report having returned to smoking at 1- or 4-month follow-up.

† Estimated percentage and number (n) of people abstinent from cigarette smoking for 6 months, after imputing the abstinence rate in those missing at follow-up. Risk ratio (RR) calculated from these estimates.
